# Supplementary material for: Supraclavicular Lymph Node Dissection in Breast Cancer with Synchronous Supraclavicular Metastases: A Systematic Review and Network Meta-Analysis
Source: Cancers (Basel). 2025 Jun 21;17(13):2081. doi: 10.3390/cancers17132081 (PMC12248947; doi:10.3390/cancers17132081)
Supplement: Supplementary file 1 [file cancers-17-02081-s001.zip › cancers-3688556-supplementary.pdf]

Supplementary Material

Table S1. Full search strategy for the various databases

PubMed

("Breast Neoplasms"[Mesh] OR "breast cancer" OR "breast carcinoma")
AND
("Supraclavicular Lymph Nodes"[Mesh] OR "supraclavicular lymph node" OR "supraclavicular metastasis" OR "ipsilateral supraclavicular" OR "synchronous supraclavicular" OR "SCV metastasis" OR "N3c")
AND
("Lymph Node Excision"[Mesh] OR "neck dissection" OR "lymph node dissection" OR "supraclavicular dissection" OR "SLND")
AND
("Radiotherapy"[Mesh] OR "radiotherapy" OR "radiation therapy" OR "RT")
AND
("Systemic Therapy" OR "chemotherapy" OR "endocrine therapy" OR "hormonal therapy" OR "targeted therapy" OR "neoadjuvant therapy" OR "adjuvant therapy" OR "ST")
AND
("Survival"[Mesh] OR "overall survival" OR "disease-free survival" OR "DFS" OR "OS")

EMBASE

('breast cancer'/exp OR 'breast carcinoma' OR 'breast neoplasm')
AND
('supraclavicular lymph node'/exp OR 'supraclavicular lymph node metastasis' OR 'ipsilateral supraclavicular' OR 'synchronous supraclavicular' OR 'n3c')
AND
('lymph node dissection'/exp OR 'neck dissection' OR 'supraclavicular dissection' OR 'SLND')
AND
('radiotherapy'/exp OR 'radiation therapy' OR 'RT')
AND
('systemic therapy' OR 'chemotherapy'/exp OR 'endocrine therapy'/exp OR 'hormonal therapy' OR 'targeted therapy'/exp OR 'neoadjuvant therapy' OR 'adjuvant therapy')
AND
('overall survival'/exp OR 'disease-free survival'/exp OR 'DFS' OR 'OS')

Scopus

TITLE-ABS-KEY ("breast cancer" OR "breast carcinoma" OR "breast neoplasm")
AND
TITLE-ABS-KEY ("supraclavicular lymph node" OR "supraclavicular metastasis" OR "ipsilateral supraclavicular" OR "synchronous supraclavicular" OR "SCV metastasis" OR "N3c")

AND
TITLE-ABS-KEY ("neck dissection" OR "lymph node dissection" OR "supraclavicular dissection" OR "SLND")
AND
TITLE-ABS-KEY ("radiotherapy" OR "radiation therapy" OR "RT")
AND
TITLE-ABS-KEY ("systemic therapy" OR "chemotherapy" OR "hormonal therapy" OR "endocrine therapy" OR "targeted therapy" OR "neoadjuvant therapy" OR "adjuvant therapy")
AND
TITLE-ABS-KEY ("overall survival" OR "disease-free survival" OR "DFS" OR "OS")

Cochrane CENTRAL

("breast cancer" OR "breast carcinoma" OR "breast neoplasm")
AND
("supraclavicular lymph node" OR "supraclavicular metastasis" OR "ipsilateral supraclavicular" OR "synchronous supraclavicular" OR "SCV metastasis" OR "N3c")
AND
("neck dissection" OR "lymph node dissection" OR "supraclavicular dissection" OR "SLND")
AND
("radiotherapy" OR "radiation therapy" OR "RT")
AND
("systemic therapy" OR "chemotherapy" OR "endocrine therapy" OR "hormonal therapy" OR "targeted therapy" OR "neoadjuvant therapy" OR "adjuvant therapy")
AND
("overall survival" OR "disease-free survival" OR "DFS" OR "OS")
